# A dataset of multiresolution functional brain parcellations in an elderly population with no or mild cognitive impairment

**DOI:** 10.1016/j.dib.2016.11.036

**Published:** 2016-11-18

**Authors:** Angela Tam, Christian Dansereau, AmanPreet Badhwar, Pierre Orban, Sylvie Belleville, Howard Chertkow, Alain Dagher, Alexandru Hanganu, Oury Monchi, Pedro Rosa-Neto, Amir Shmuel, John Breitner, Pierre Bellec, for the Alzheimer׳s Disease Neuroimaging Initiative

**Affiliations:** a McGill University, Montreal, QC, Canada; b Douglas Mental Health University Institute, Research Centre, Montreal, QC, Canada; c Centre de recherche de l’institut universitaire de gériatrie de Montréal, QC, Canada; d Université de Montréal, QC, Canada; e University of Calgary, AB, Canada; f Hotchkiss Brain Institute, Calgary, AB, Canada

## Abstract

We present group eight resolutions of brain parcellations for clusters generated from resting-state functional magnetic resonance images for 99 cognitively normal elderly persons and 129 patients with mild cognitive impairment, pooled from four independent datasets. This dataset was generated as part of the following study: *Common Effects of Amnestic Mild Cognitive Impairment on Resting-State Connectivity Across Four Independent Studies* (Tam et al., 2015) [1]. The brain parcellations have been registered to both symmetric and asymmetric MNI brain templates and generated using a method called bootstrap analysis of stable clusters (BASC) (Bellec et al., 2010) [2]. We present two variants of these parcellations. One variant contains bihemisphereic parcels (4, 6, 12, 22, 33, 65, 111, and 208 total parcels across eight resolutions). The second variant contains spatially connected regions of interest (ROIs) that span only one hemisphere (10, 17, 30, 51, 77, 199, and 322 total ROIs across eight resolutions). We also present maps illustrating functional connectivity differences between patients and controls for four regions of interest (striatum, dorsal prefrontal cortex, middle temporal lobe, and medial frontal cortex). The brain parcels and associated statistical maps have been publicly released as 3D volumes, available in .mnc and .nii file formats on figshare and on Neurovault. Finally, the code used to generate this dataset is available on Github.

**Specifications Table**

**Table:** 

Subject area	*Biology*
More specific subject area	*Neuroscience*
Type of data	*Images*
How data was acquired	*MRI, resting-state functional MRI (Philips & Siemens 3 T scanners)*
Data format	*Analyzed*
Experimental factors	*Pre-processing for motion-related or other artifacts, group-level statistical analysis*
Experimental features	*We pooled resting-state fMRI data from 4 independent studies with cognitively normal elderly subjects and patients with mild cognitive impairment to generate 1) group-level functional brain parcellations with varying numbers of parcels, and 2) maps illustrating functional connectivity differences between patients and controls in four parcels of interest.*
Data source location	*Canada & The United States*
Data accessibility	*Data is within this article and available online at the following sites: Figshare:*http://dx.doi.org/10.6084/m9.figshare.1480461*Neurovault:*http://neurovault.org/collections/1003/*Github:*https://github.com/SIMEXP/mcinet

**Value of the data**

• These parcellations can be used as atlases for brain imaging studies in elderly populations.
• The functional clusters and *t*-maps we have derived can be used as target regions in hypothesis-driven studies, especially for those interested in aging, mild cognitive impairment and dementia.
• The code can be adapted to generate similar atlases on other datasets or populations.

## 1 Data

This data release contains group brain parcellations at multiple resolutions (4, 6, 12, 22, 33, 65, 111, and 208 parcels) generated from resting-state functional magnetic resonance images for 99 cognitively normal elderly persons and 129 patients with mild cognitive impairment. This work also includes parcellations that contain regions-of-interest (ROIs) that are spatially connected and span only one hemisphere at 8 resolutions (10, 17, 30, 51, 77, 137, 199, and 322 total ROIs). Labels based on typical resting-state networks, and their decomposition into subnetworks or regions, are proposed for all brain parcels. This release also includes unthresholded maps of connectivity differences (*t*-maps) between patients and controls for four seeds/regions of interest (striatum, dorsal prefrontal cortex, middle temporal lobe, and medial frontal cortex).

## 2 Experimental design, materials and methods

### 2.1 Participants

We pooled resting-state functional magnetic resonance imaging (fMRI) data from four independent studies: the Alzheimer׳s Disease Neuroimaging Initiative 2 (ADNI2) sample, two samples from the Centre de recherche de l’institut universitaire de gériatrie de Montréal (CRIUGMa and CRIUGMb), and a sample from the Montreal Neurological Institute (MNI) [3]. All participants gave their written informed consent to engage in these studies, which were approved by the research ethics board of the respective institutions, and included consent for data sharing with collaborators as well as secondary analysis. Ethical approval was also obtained at the site of secondary analysis (CRIUGM).

The ADNI2 data used in the preparation of this article were obtained from the Alzheimer׳s Disease Neuroimaging Initiative (ADNI) database (adni.loni.usc.edu). ADNI was launched in 2003 by the National Institute on Aging, the National Institute of Biomedical Imaging and Bioengineering, the Food and Drug Administration, private pharmaceutical companies and non-profit organizations, as a $60 million, 5-year public-private partnership representing efforts of co-investigators from numerous academic institutions and private corporations. ADNI was followed by ADNI-GO and ADNI-2 that included newer techniques. Subjects included in this study were recruited by ADNI-2 from all 13 sites that acquired resting-state fMRI on Philips scanners across North America. For up-to-date information, see www.adni-info.org.

The final combined sample included 112 cognitively normal elderly subjects (CN) and 143 patients with mild cognitive impairment (MCI). In the CN group, the mean age was 72.0 (s.d. 7.0) years, and 38.4% were men. Mean age of the MCI subjects was 72.7 (s.d. 7.7) years, and 50.3% were men. For more information about recruitment or participant characteristics, please refer to Tam et al. [1].

### 2.2 Imaging data acquisition

All resting-state fMRI and structural scans were acquired on Philips and Siemens 3 T scanners. For more detailed information on the imaging parameters, please refer to Tam et al. [1].

### 2.3 Computational environment

All experiments were performed using the NeuroImaging Analysis Kit (NIAK)^1^[4] version 0.12.18, under CentOS version 6.3 with Octave^2^ version 3.8.1 and the Minc toolkit^3^ version 0.3.18. Analyses were executed in parallel on the “Guillimin” supercomputer,^4^ using the pipeline system for Octave and Matlab [5], version 1.0.2. The scripts used for processing can be found on Github.^5^

### 2.4 Pre-processing

Each fMRI dataset underwent preprocessing as described in Tam et al. [1]. A more detailed description of the pipeline can also be found on the NIAK website^6^ and Github.^7^

### 2.5 Parcellation of the brain into functional clusters

**Fig. 1:**
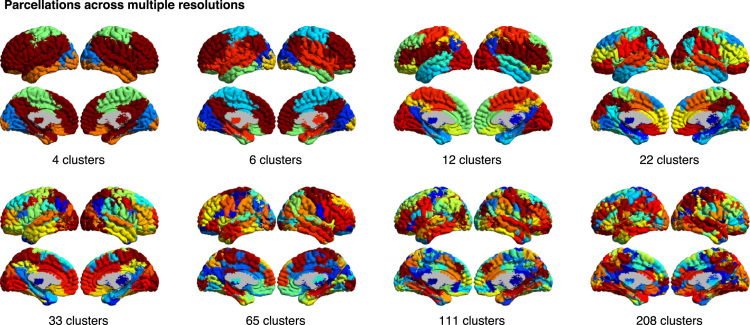
Functional parcellations across resolutions (or number of clusters).

**Fig. 2:**
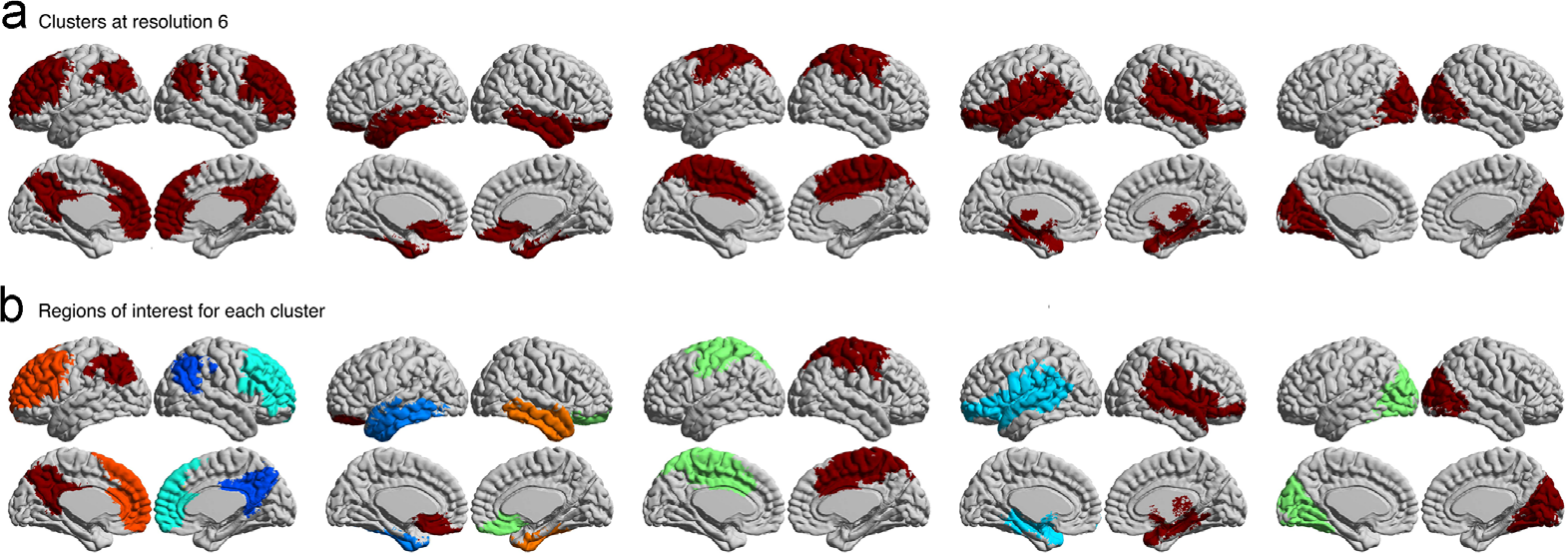
Clusters at resolution 6 (cerebellum not shown) and their respective regions-of-interest. Note how each cluster in (A) is bihemispheric prior to breaking down into multiple spatially constrained regions-of-interest in (B).

After pre-processing, we generated functional brain atlases at eight resolutions with a bootstrap analysis of stable clusters [2], containing 4, 6, 12, 22, 33, 65, 111 and 208 total parcels, as described in Tam et al. [1]. These eight resolutions of brain parcellations (Fig. 1), registered to both symmetric and asymmetric MNI templates, have been released on figshare^8^ and Neurovault.^9^ These eight resolutions were further processed to generate eight parcellations that contain ROIs that are spatially connected and span only one hemisphere (for an example, see Fig. 2). These latter parcellations contain 10, 17, 30, 51, 77, 137, 199, and 322 total ROIs.

**Fig. 3:**
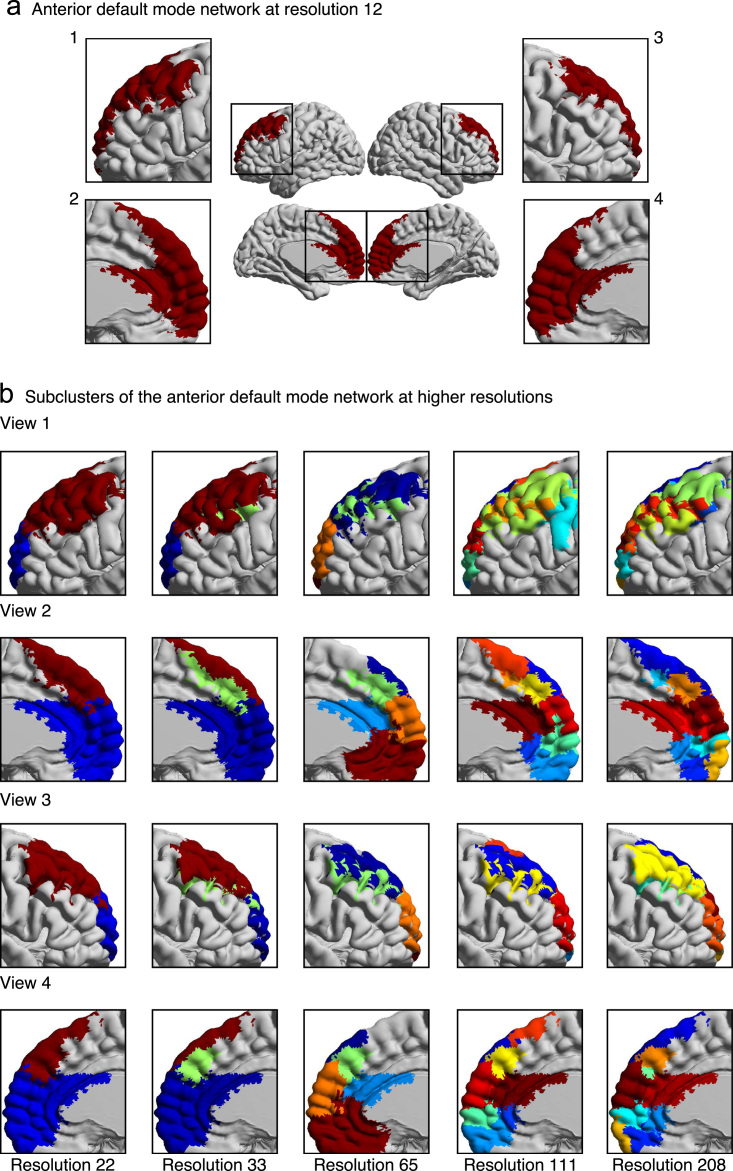
The decomposition of the anterior default mode network into smaller subclusters at higher resolutions in four different views. Resolution 12 was used as a reference for the labeling of subnetworks at higher resolutions.

We have provided labels for each parcel at every resolution, except for resolutions 4 and 6 due to the merging of networks at those low resolutions. At resolution 4, we observed the sensory-motor network, visual network, a network that resembles the endogenous network [6] and a network that merges the cerebellum and the mesolimbic network together. At resolution 6, we observed the visual network, cerebellum, mesolimbic network, sensory-motor network, a network that merges the deep gray matter nuclei with the frontoparietal network, and a network that merges the default mode network with the posterior attention network. For resolution 12, we manually labeled each parcel (deep gray matter nuclei (DGMN), posterior default mode network (pDMN), medial temporal lobe (mTL), ventral temporal lobe (vTL), dorsal temporal lobe (dTL), anterior default mode network (aDMN), orbitofrontal cortex (OFC), posterior attention (pATT), cerebellum (CER), sensory-motor (SM), visual (VIS), and frontoparietal network (FPN)). Then, we decomposed the networks at resolution 12 into smaller subclusters at all higher resolutions (for an example, see Fig. 3). Each parcel at higher resolutions was labeled in reference to the parcels at resolution 12, with the following convention: (resolution)_(parcel label)_(#); for example, at resolution (R) 22, the anterior default mode splits into two clusters, which were named “R22_aDMN_1” and “R22_aDMN_2”.

### 2.6 Derivation of functional connectomes

Between and within-clusters connectivity was measured as described in Tam et al. [1].

### 2.7 Statistical testing

**Fig. 4:**
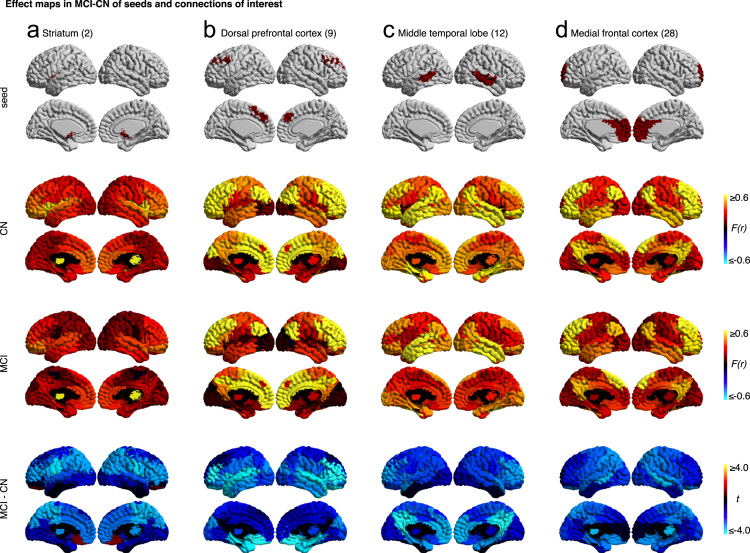
Maps for a selection of four seeds that show effects related to MCI at resolution 33. These effect maps reveal the spatial distribution of the differences in functional connectivity for (A) striatum, (B) dorsal prefrontal cortex, (C) middle temporal lobe, and (D) the medial frontal cortex. For each panel, the top line maps the spatial location of the seed region in red, the second and third lines show the connectivity (Fisher-transformed correlation values *(F(r))* between the designated seed region and the rest of the brain in CN and MCI respectively, and the fourth line shows a difference map between MCI and CN (*t*-test). The numbers in parentheses refer to the numerical IDs of the clusters in the 3D parcellation volume at resolution 33.

To test for differences between aMCI and CN at a resolution of 33 clusters, we used a general linear model (GLM) for each connection between two parcels [7]. Specific details of the GLM can be found in Tam et al. [1]. From this analysis, we present uncorrected *t*-maps illustrating functional connectivity differences between patients and controls for four seeds/regions of interest (striatum, dorsal prefrontal cortex, middle temporal lobe, and medial frontal cortex) (Fig. 4). These maps have been released on figshare and Neurovault. These four seeds were chosen for further analyses because, together, they were associated with 47% of all significant group differences across all brain regions. Briefly, we found that MCI patients exhibited reduced connectivity between default mode network nodes and between areas of the cortico-striatal-thalamic loop. For a more in-depth presentation and discussion of results, please refer to Tam et al. [1].

## Footnotes

1 http://simexp.github.io/niak/.

2 https://www.gnu.org/software/octave/.

3 http://www.bic.mni.mcgill.ca/ServicesSoftware/ServicesSoftwareMincToolKit.

4 http://www.calculquebec.ca/en/resources/compute-servers/guillimin.

5 https://github.com/SIMEXP/mcinet.

6 http://niak.simexp-lab.org/pipe_preprocessing.html.

7 https://github.com/SIMEXP/mcinet/tree/master/preprocess.

8 http://dx.doi.org/10.6084/m9.figshare.1480461.

9 http://neurovault.org/collections/1003/.
